# Complementary and Alternative Medicine for Diseases and Disorders in Digestive Tract: Basic to Clinics

**DOI:** 10.1155/2013/565279

**Published:** 2013-10-24

**Authors:** Chang Gue Son, Zhao Xiang Bian, Jing Hua Wang, H. Balaji Raghavendran

**Affiliations:** ^1^Liver & Immunology Research Center, Daejeon Oriental Hospital of Daejeon University, Daejeon 301-724, Republic of Korea; ^2^School of Chinese Medicine, Hong Kong Baptist University, Hong Kong; ^3^Key Laboratory of Xin'an Medicine, Ministry of Education, Clinical College of TCM, Anhui University of TCM, Hefei, Anhui Province 230011, China; ^4^Department of Orthopaedic Surgery, Faculty of Medicine, University of Malaya, 50603 Kuala Lumpur, Malaysia

The gastrointestinal (GI) tract is a long pathway of about 9 m, passing through the longitudinal center of the body. The disorders or diseases of GI tract are very commonly observed in clinical practices. As conventional medical therapies either do not produce satisfactory results or may have side effects, many patients seek complementary and alternative medicine (CAM) [1]. Furthermore, patients prefer additional CAM therapies to improve health-related quality of life through holistic concepts [2]. In the USA, it has been reported that 51% of the patients with GI tract disorders have tried some form of CAM [3], whereas, in the UK, 26% of the patients with GI tract symptoms and 48% of the patients with irritable bowel syndrome (IBS) have been noted to use CAM [4].

With the increasing numbers of patients and practitioners using CAM modalities, the number of studies on the application of CAM for the treatment of GI disorders has increased. CAM modalities include a wide variety of approaches, including acupuncture, moxibustion, herbal medicine, nutrition, microbial therapy using probiotics, meditation, chiropractic, cupping, massage, yoga, and Qigong. Among them, herbal medicine (single herb or mixture of multiple herbs) and acupuncture have been most extensively studied for GI disorders [5]. For example, clinical studies in China have indicated that several herbal drugs show superior effect to western drugs in the management of ulcerative colitis, a refractory and chronic inflammatory bowel disease (IBD) [6, 7]. In particular, functional GI disorders, such as functional dyspepsia and IBS, have been relatively main targets of study using acupuncture or moxibustion [8, 9]. Serial trials have proposed that modulation of GI motility by acupuncture could be one of the mechanisms responsible for its effects on functional disorders in the GI track [10, 11]. However, we still need to prove the efficacy, safety, and cost-effectiveness of CAM treatments for GI illnesses.

This special issue is an attempt to contribute to the knowledge on CAM treatments for GI diseases and disorders. We particularly called for articles that have explored the clinical or animal-based evidence demonstrating the effectiveness of CAM. A collection of seven original research articles and two reviews are presented, which address the clinical evaluation and animal-based pharmacological effects of herbal drugs on GI disorders, as well as the central neural mechanisms of acupuncture in the regulation of gastric motility. The two review articles have systematically analyzed the clinical benefits of two traditional Chinese herbal formulas on diabetic gastroparesis and functional dyspepsia. Interestingly, two research articles have simultaneously reported the clinical results of an identical herbal drug (*Ban Xia Xie Xin Tang* in China and *Banha-Sasim-Tang* in Korea) used for the treatment of functional dyspepsia, through randomized controlled trial (RCT). Furthermore, the subjects of five animal studies were colorectal cancer, peptic ulcer, colitis, and liver injury, and their treatments comprised various multiple herbal medicines including single herb.

This special issue provides valuable information to practitioners and researchers working in the field of GI tract. However, there are still some major challenges due to the lack of convincing clinical evidence and less standardized therapeutics of CAM for digestive tract problems. Accordingly, further evidence-based clinical trials should be developed and implemented.
